# Mitochondria-Targeting Metal Complexes: Design Principles, Mechanisms of Action, and Translational Perspectives

**DOI:** 10.3390/biom16070987

**Published:** 2026-07-04

**Authors:** Donatella Coradduzza, Giacomo Senzacqua, Rosita Cappai, Serenella Medici

**Affiliations:** 1 Department of Biomedical Sciences, University of Sassari, 07100 Sassari, Italy; dcoradduzza@uniss.it; 2 Department of Chemical, Physical, Mathematical and Natural Sciences, University of Sassari, 07100 Sassari, Italy; giacomo.senzacqua@gmail.com (G.S.); rcappai1@uniss.it (R.C.)

**Keywords:** mitochondria, metal complexes, metallodrugs, apoptosis, reactive oxygen species, electron transport chain, bioinorganic chemistry, theranostics, ΔΨm, ICP-MS, speciation, clinical safety monitoring, translational failure

## Abstract

Mitochondria-targeting metal complexes (MTMCs) are a mechanistically distinct class of metallopharmaceuticals. Unlike first-generation platinum drugs that form nuclear DNA adducts, MTMCs exploit organelle-specific vulnerabilities such as hyperpolarised mitochondrial membrane potential (ΔΨm), elevated reactive oxygen species (ROS), limited mitochondrial DNA (mtDNA) repair capacity, and redox-dependent enzymes such as thioredoxin reductase (TrxR). We systematically searched PubMed, Web of Science, Scopus, and Google Scholar databases for studies published between 2016 and 2026, applying predefined inclusion criteria that included subcellular localization evidence and functional bioenergetic endpoints. The search identified 147 studies covering Pt(II/IV), Ru(II/III), Au(I/III), Ir(III), Os(II), Re(I), and V(IV/V) complexes and metal–organic framework nanoplatforms. Mechanistic evidence converges on four intramitochondrial target categories: inhibition of ETC (Electron Transport Chain) Complexes I/III with consequent ATP depletion; ROS overproduction, coupled with glutathione and TrxR depletion; outer mitochondrial membrane permeabilization and intrinsic apoptotic cascade activation; and mtDNA damage within a compartment limited to base excision repair. Multi-modal cell death—the co-occurrence of apoptosis, ferroptosis, necroptosis, and autophagic cell death—was a recurrent finding across the reviewed studies. This review thoroughly surveys the latest trends in MTMC drug design (metals, ligand structures, and mechanisms of action) and summarises analytical techniques for speciation, pharmacokinetics, safe monitoring, and resistance, while critically analysing translational barriers and clinical failures. To address the field’s inconsistent terminology, we introduce an explicit localization evidence hierarchy that distinguishes mitochondria-targeting complexes (through quantitative ICP-MS fractionation or co-localization with defined Pearson/Manders coefficients) from simply mitochondria-localising or mitochondria-perturbing agents, and we apply it throughout. We also point out that the idea of selectivity being purely driven by membrane voltage (ΔΨm) and thermodynamics is constrained by membrane and protein binding, as well as the transmembrane pH gradient, kinetic limitations, and demonstrated heterogeneity of cancer-cell membrane potential, and, as such, the functional mitochondrial effects must not be equated with mitochondrial accumulation. Since elemental quantification cannot distinguish intact complex from protein adducts and decomposition products, speciation-aware pharmacokinetics emerges as a prerequisite for a credible exposure–response interpretation. The translational progress will depend less on new chemotypes than on this analytical and pharmacokinetic rigour, together with organelle-level safety monitoring and biomarker-guided patient selection.

## 1. Introduction

### 1.1. Mitochondria as Central Hubs in Cellular Physiology

Mitochondria are double-membraned organelles of endosymbiotic origin, long dubbed the “powerhouses of the cell” for their central role in generating most cellular adenosine triphosphate (ATP) [1]. This crucial function in energy metabolism, particularly in the fast-growing environment of cancer cells, makes them an optimal target for new types of drugs [2].

Targeting mitochondria is an attractive strategy in cancer therapy for several reasons: (1) they play key roles in cell death: mitochondria are central to initiating several programmed cell death pathways, including apoptosis (the most common form), as well as autophagy and ferroptosis [3,4,5,6,7,8]. Destroying them ensures the cell death signal is both strong and difficult for cancer cells to bypass. (2) They can help overcome drug resistance: many cancer cells become resistant to traditional drugs, like cisplatin, which work by damaging nuclear DNA. By targeting mitochondria instead, these metal complexes often prove effective against otherwise drug-resistant cancers [9,10,11,12].

As mentioned above, mitochondria generate chemical energy; in fact, they are the principal site of oxidative phosphorylation (OXPHOS), where reduced substrates are converted into ATP through the electron transport chain—composed of Complexes I–IV embedded in the inner mitochondrial membrane (IMM) [13,14,15,16]. The chemiosmotic coupling of electron flux to proton translocation involved in this process generates a mitochondrial membrane potential (ΔΨm) that, in metabolically active cells, ranges from approximately −150 to −180 mV under resting State 4 conditions in isolated mitochondria [17,18,19,20]. However, calibrated measurements in intact cells consistently report a ΔΨm between −128 and −145 mV. This discrepancy carries direct pharmacological implications [21], which will be addressed in Section 10. Beyond bioenergetics, mitochondria integrate intrinsic apoptotic signalling through BCL-2 (B cell lymphoma 2) family proteins at the outer mitochondrial membrane (OMM). They also constitute the cell’s primary intracellular source of reactive oxygen species (ROS), which arise as obligate byproducts of electron flux through Complexes I and III [22,23].

Mitochondrial dysfunction is now understood as a mechanistic driver across oncology, neurodegeneration, and metabolic disease rather than a mere epiphenomenon [5,24,25,26]. In cancer, the Warburg effect—the preferential use of aerobic glycolysis over oxidative phosphorylation even when oxygen is abundant—is associated with elevated steady-state ROS, altered ETC stoichiometry, BCL-2 overexpression, and somatic mtDNA mutations [27,28]. Together, these features create a pharmacological vulnerability that is susceptible to organelle-directed intervention. This vulnerability forms the mechanistic foundation of the MTMC therapeutic strategy [27,29].

### 1.2. Limitations of the Cisplatin Paradigm and the Rationale for Mitochondrial Targeting

**Figure 1:**
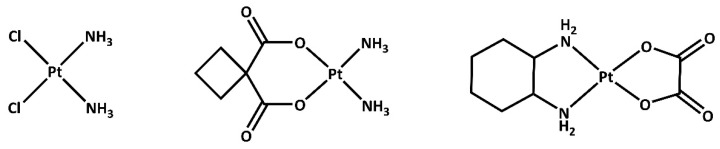
Cisplatin and its derivatives carboplatin and oxaliplatin.

First-generation clinical metallodrugs (e.g., cisplatin, carboplatin, and oxaliplatin; Figure 1) exert cytotoxicity primarily by forming nuclear DNA adducts at the d(GpG) and d(ApG) sequences [30,31,32,33]. This mechanism is constrained by five resistance pathways: (i) reduced cellular accumulation via CTR1 (high affinity copper uptake protein 1) downregulation; (ii) enhanced nucleotide excision repair (NER) through ERCC1/XPC upregulation; (iii) mismatch repair (MMR) deficiency, which enables adduct tolerance; (iv) BCL-2/BCL-XL (B-cell lymphoma-extra-large) overexpression blocking downstream outer mitochondrial membrane permeabilization (MOMP); and (v) GSH-mediated drug deactivation. Resistance mediated by pathways (i), (ii), and (iii) is bypassed by MTMCs, which act through the mitochondrial compartment independently of nuclear DNA damage recognition and NER-dependent repair. Instead, mitochondrial accumulation follows an electrophoretic mechanism governed by the Nernst equation [30,34,35,36,37,38]. This process is driven by biophysical forces rather than carrier-mediated transport, thereby conferring mechanistic independence from CTR1-based resistance. Such theoretical advantages must nevertheless be reconciled with the translational barriers documented in the following sections [39].

## 2. Methodology

### 2.1. Systematic Search Strategy

A structured bibliographic search was conducted between February and April 2026 across PubMed/MEDLINE, Web of Science Core Collection, Scopus, and Google Scholar. The principal search strategy combined four thematic blocks using Boolean operators. First, we used variations of “mitochondria-target” OR “mitochondrial accumulation” OR “ΔΨm” OR “mtDNA” combined with “metal complex” OR “metallodrug” OR specific metal terms (platinum, ruthenium, iridium, rhenium, gold, osmium, technetium, copper-64, or gallium-68) and functional keywords (“OXPHOS”, “electron transport chain”, “ROS”, “glutathione”, “TCA”, “apoptosis”, “mitophagy”, “ferroptosis”, “necroptosis”). Second, we applied filters for targeting moieties (triphenylphosphonium, TPP, MPP, and FMTS). Third, we included theranostic terms (FLIM, PLIM, ^99^^m^Tc-MIBI, and ^68^Ga-TPP). Fourth, we incorporated translational failure terms (NAMI-A, KP1019, titanocene, and “clinical failure metal complex”).

### 2.2. Inclusion and Exclusion Criteria

Inclusion required the following: a peer-reviewed publication in English (January 2016–April 2026), a metal complex with defined coordination chemistry and spectroscopic characterisation, subcellular localisation evidence (ICP-MS fractionation or co-localisation, Pearson r ≥ 0.80 with MitoTracker), and at least one functional bioenergetic endpoint (OCR, ΔΨm, ROS, or ATP). Studies reporting only secondary mitochondrial dysfunction, non-peer-reviewed sources, and compounds lacking mechanistic characterisation of organelle-level engagement were excluded. A total of 147 studies met criteria after deduplication and full-text screening.

Throughout this review, we differentiate three operationally distinct categories: (i) mitochondria-targeting complexes, for which preferential mitochondrial accumulation is directly demonstrated by subcellular ICP-MS fractionation or by quantitative co-localization with mitochondria-selective probes (Pearson r ≥ 0.80 and/or Manders’ coefficients); (ii) mitochondria-localising complexes, for which qualitative imaging evidence of mitochondrial residence exists in the absence of quantitative accumulation data; (iii) mitochondria-perturbing complexes, which alter mitochondrial function (ΔΨm, OCR, ROS) without demonstrated organelle-selective accumulation. Only categories (i) and (ii) are treated as mitochondria-targeting, while category (iii) compounds, including the antimetastatic agent NAMI-A, are discussed as mechanistic comparators rather than classified as mitochondria-targeting agents. Each complex tabulated in this review is accompanied by an explicit indication of the localisation evidence underpinning its classification.

### 2.3. Use of AI

ChatGPT OpenAI’s ChatGPT-5 was employed to correct grammar, punctuation, and typos, but also for language improvement.

## 3. Chemical Design Principles

### 3.1. The Thermodynamic Basis of Mitochondrial Accumulation

**Figure 2:**
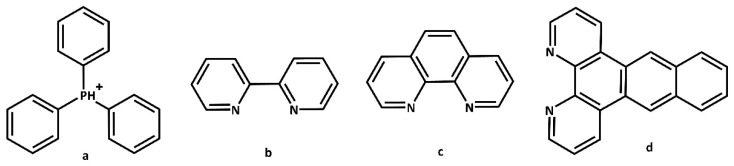
Triphenylphosphonium anion (TPP^+^, **a**) and some polypyridyl ligands: bipyridine (bipy, **b**), o-phenanthroline (phen, **c**), and dipyridophenazine (dppz, **d**).

The mitochondrial accumulation of lipophilic cations is commonly rationalised by the Nernst equation, which predicts that, for a monovalent cation at 37 °C, each 61.5 mV increase in membrane potential corresponds to an approximately ten-fold concentration gradient at the electrochemical equilibrium [40,41,42,43]. Based on the combined contribution of the plasma membrane potential (Δψp ∼ −40 mV) and the mitochondrial one (ΔΨm ∼ −170 mV), theoretical intramitochondrial-to-extracellular enrichment factors of several hundred- to several thousand-fold have been proposed for monovalent lipophilic cations [44,45,46]. However, these values represent ideal thermodynamic predictions and do not fully account for additional determinants of intracellular distribution, including membrane permeability, molecular size, protein binding, and kinetic non-equilibrium effects. In this context, triphenylphosphonium ion (TPP^+^) (Figure 2) remains the most extensively validated mitochondriotropic vector. However, its intrinsic uncoupling activity at concentrations achieved after high-dose administration poses a safety liability, which mandates carrier-only control experiments in all mechanistic studies [47,48,49].

This Nernstian description is, however, an idealised thermodynamic equilibrium prediction, and the biological reality of intracellular distribution departs from it in several quantitatively decisive ways. First, the premise that cancer cells possess a uniformly elevated ΔΨm—and are therefore intrinsically selective targets for lipophilic cations—is not supported by any modern, absolute-millivolt-calibrated measurements: single-cell TMRM calibration shows that ΔΨm is in fact more heterogeneous within cancer-cell populations than in matched non-malignant cells and is governed predominantly by intramitochondrial factors, with individual cancer cells spanning a broad range of potentials and median values that are well below their own maximal ΔΨm [50]. Selectivity arguments resting solely on a tumour-versus-normal potential differential are therefore probabilistic rather than deterministic. Second, a substantial fraction of accumulated cation is not free in the matrix but bound to the inner-membrane surface and to matrix proteins, inflating the apparent uptake and, if not corrected, it leads to systematic overestimation of ΔΨm; additionally, uptake depends on plasma- and tissue-protein binding, which lowers the free fraction available for the electrophoretic partitioning, and on intracellular sequestration and trafficking that impose non-equilibrium constraints. Third, the relationship is kinetically bounded: Nernstian behaviour holds for lipophilic dications only at low levels of accumulation and breaks down above a threshold potential [41]. Finally, the targeting cation is not a passive spectator—methyltriphenylphosphonium inhibits 2-oxoglutarate dehydrogenase with a low-millimolar IC_50_ that corresponds to pharmacologically relevant effects given the large accumulation ratio [51]—although rational modification of the phosphonium aryl rings can abolish uncoupling without compromising delivery [47]. Thermodynamic accumulation models should therefore guide design but cannot, on their own, establish either mitochondrial selectivity or organelle-specific engagement, both of which require the direct localization evidence defined in Section 6.

Importantly, this classical thermodynamic model was primarily derived from experiments on isolated mitochondria under State 4 (resting) conditions, where ΔΨm approaches −180 mV, rather than from calibrated intact-cell measurements. As addressed in Section 10, intact-cell TMRM-based calibration studies in living cells generally report ΔΨm values in cancer cells between −128 and −140 mV—substantially lower than those measured in isolated mitochondria and not consistently higher than those observed in corresponding normal cells. These findings challenge the assumption that tumour selectivity can be explained solely by a larger ΔΨm differential [17,52].

### 3.2. Beyond TPP^+^: Emerging Mitochondrial Targeting Strategies

Although the triphenylphosphonium cation remains the most extensively validated mitochondriotropic vector, its intrinsic liabilities—chain-length-dependent uncoupling of oxidative phosphorylation, inhibition of respiratory complexes, and inhibition of 2-oxoglutarate dehydrogenase at the elevated intramitochondrial concentrations produced by electrophoretic accumulation [53,54]—have motivated a second generation of targeting strategies that engage the organelle through orthogonal mechanisms. These approaches are increasingly relevant to metallodrug design, where the targeting moiety must be reconciled with the coordination sphere, redox behaviour, and speciation of the metal centre.

In this respect, peptide-based vectors are the most mechanistically distinct alternative. The mitochondria-penetrating peptides (MPPs) developed by Kelley and co-workers interleave the cationic D-arginine and the lipophilic cyclohexylalanine residues, combining charge and hydrophobicity to traverse the inner membrane while resisting proteolysis; their modular sequence allows tuning of charge, lipophilicity, and cargo capacity without the uncoupling liability of alkyl-TPP^+^ chains [54]. The Szeto–Schiller tetrapeptide SS-31 (elamipretide; D-Arg-2′,6′-Dmt-Lys-Phe-NH_2_) operates by a fundamentally different principle: rather than responding to the membrane potential, it accumulates by binding the anionic inner-membrane phospholipid cardiolipin, thus stabilising cardiolipin–cytochrome c interactions and preserving the cristae architecture [55,56]. This ΔΨm independence is a decisive conceptual advantage for tumours in which the membrane potential is depolarised or heterogeneous, and SS-31 provides the field’s first regulatory proof-of-concept, having received an FDA accelerated approval in September 2025. Cardiolipin-directed targeting is attractive mainly because cardiolipin is largely confined to the inner mitochondrial membrane and is externalised during mitochondrial stress, conferring a degree of disease selectivity that potential-driven cations inherently lack.

A parallel strategy seeks selectivity not in the vector but in the trigger. Stimuli-responsive delivery systems mask the targeting cation or cytotoxic metallofragment until it encounters a tumour- or organelle-specific signal. ROS-responsive linkers (e.g., thioketal or aryl-boronate bonds) exploit the elevated mitochondrial ROS flux to release payloads selectively, as demonstrated for TPP^+^-thioketal conjugates of metabolic inhibitors [57]; glutathione-responsive disulfide linkers are cleaved in the reducing intracellular and intramitochondrial milieu; pH-responsive systems exploit the acidic tumour microenvironment to unmask cationic charge only after extravasation; and photoactivated metal complexes—including Ru(II) and Pt(IV) prodrugs—restrict cytotoxicity to the irradiated field, adding spatial control to organelle targeting [58]. These designs reduce the systemic cationic toxicity and off-target accumulation that limit constitutively charged vectors, at the cost of greater synthetic complexity and a dependence on the spatial reliability of the triggering signal.

Dual- and multi-targeting constructs represent the most integrated approach, pairing an organelle-directing element with a tissue- or biomarker-selective ligand so that tumour selectivity and organelle selectivity are imposed sequentially. Compared with TPP^+^, each of these strategies trades the simplicity and predictable ΔΨm-driven accumulation of the phosphonium cation for either a more benign bioactivity profile (peptides, cardiolipin binders), conditional activation (stimuli-responsive systems), or layered selectivity (dual targeting). None has yet matched the depth of mechanistic characterisation available for TPP^+^ conjugates, and only the cardiolipin-targeting peptide class has reached regulatory approval—for a mitochondrial myopathy rather than oncology. For metallodrugs specifically, the central design challenge is that the targeting strategy must survive the aquation, ligand-exchange, and protein-binding processes that reshape the complex in circulation (Section 8); a vector validated on a luminescent reporter does not guarantee an equivalent behaviour when conjugated to a kinetically labile metal centre. These strategies should therefore be regarded as complementary to, rather than replacements for, the phosphonium paradigm, with selection dictated by the metal, its redox chemistry, and the intended clinical context.

### 3.3. Ligand Engineering and Redox Tuning

The use of proper ligands is essential in designing effective metallodrugs, as summarised in Table 1. Polypyridyl ligands (bpy, phen, dppz, and their extended analogues; Figure 2) confer remarkable lipophilicity and planar aromatic character, which favour membrane partitioning and mitochondrial accumulation for Ru(II) and Ir(III) complexes without requiring TPP^+^ conjugation. N-heterocyclic carbene (NHC) ligands, instead, contribute to metabolic stability through strong metal–carbon σ-bonds and facilitate cellular uptake [59,60,61,62,63,64].

**Scheme 1:**
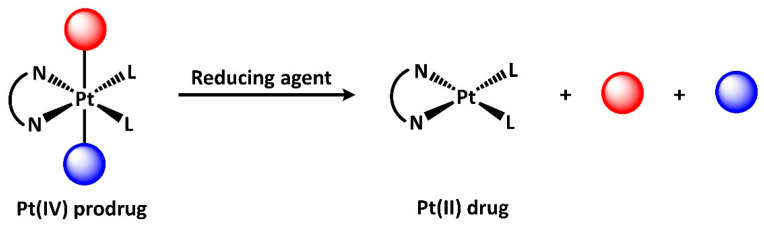
Activation by reduction of Pt(IV) prodrugs.

For prodrug strategies, the higher oxidation state stability of Pt(IV) relative to Pt(II) in plasma can be exploited to improve systemic stability. Pt(IV) complexes are normally converted into Pt(II) active species through activation by reduction pathways (Scheme 1) [65,66,67]. Bioreductive activation occurs in the reducing intramitochondrial milieu (NADPH/NADH redox window: approximately −0.3 to −0.1 V vs. NHE), enabling selective release of Pt(II) together with the axial ligand payloads at the target site [74,75,76].

Table 2 provides a comparative overview of eleven representative MTMCs drawn from the 2016–2026 literature, spanning seven metal series. A detailed mechanistic discussion follows in the next sections; key data are contextualised within the translational failure framework developed in Section 10.

## 4. Metal-Specific Design and Mechanistic Case Studies

### 4.1. Platinum Complexes

**Figure 3:**
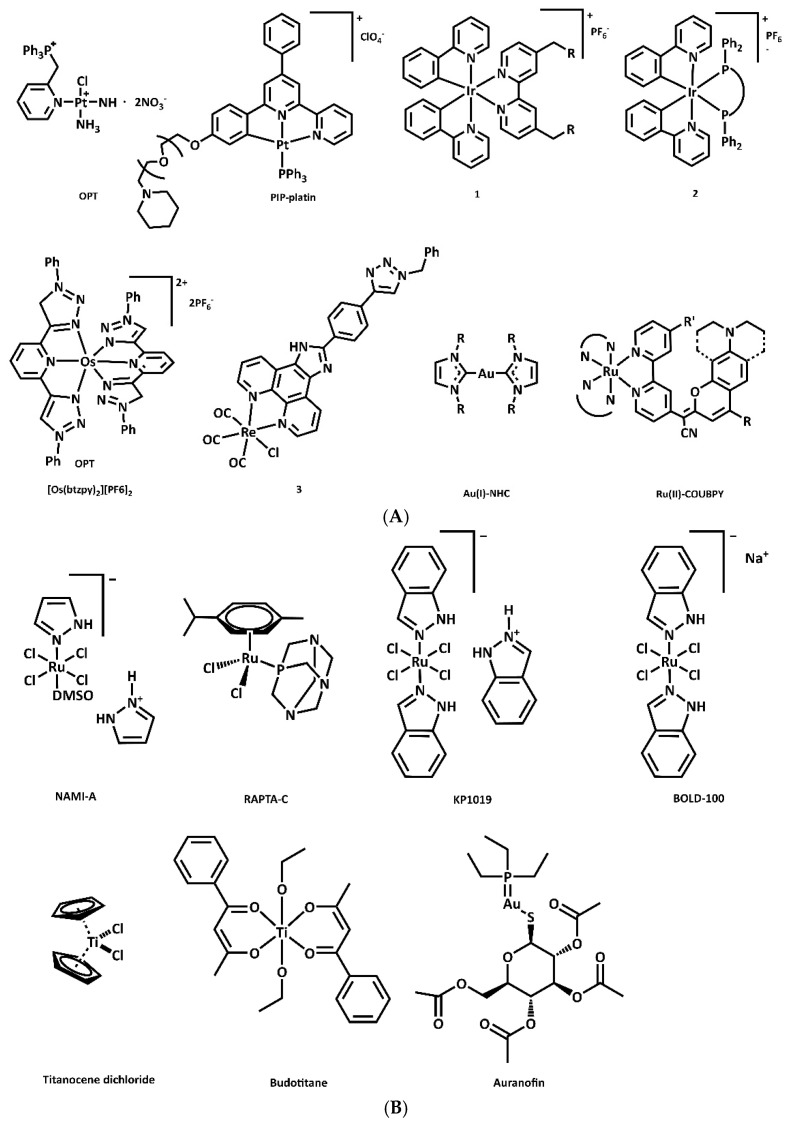
(**A**) Validated mitochondria-targeting metal complexes (tier (i)/(ii) of Section 6 hierarchy). Structures of complexes with quantitative localisation evidence: OPT (Pt; ICP-MS fractionation) [79]; PIP-platin (Pt; ICP-MS fractionation) [77]; **1**, mitochondria-immobilised cyclometalated Ir(III) (Pearson 0.74–0.87 + ICP-MS) [93]; **2**, Ir(III) viscosity probe (Pearson 0.93) [94]; Os(II) triazole (Pearson 0.85) [95]; **3**, Re(I) tricarbonyl (Pearson up to 0.918) [83]; Au(I)–NHC (mitochondrial accumulation + TrxR inhibition) [71]; and Ru(II)–COUBPY (Pearson 0.80; Manders M1 0.59/M2 0.78) [60]. (**B**) Clinically investigated metallodrugs and mechanistic comparators (tier (III) or non-mitochondrial primary target; shown for translational context, not as targeting exemplars): cisplatin/carboplatin/oxaliplatin (nuclear DNA adducts); NAMI-A (antimetastatic; mitochondrion-perturbing, no accumulation demonstrated); KP1019/BOLD-100 (GRP78/ER-stress, ~98–99% albumin-bound); RAPTA-C (antimetastatic comparator); titanocene dichloride and budotitane (clinical failure comparators, rapid hydrolysis); auranofin (TrxR inhibition; intact parent undetectable in blood).

PIP-platin (Li et al., 2017 [77]) is a cyclometalated cationic Pt(II) complex (Figure 3A) whose intrinsic positive charge drives mitochondrial accumulation, confirmed by subcellular ICP-MS fractionation. It elicits ROS elevation, ΔΨm collapse, and cytochrome c release, with concomitant disruption of β-catenin trafficking that impairs tumour-cell migration and invasion. For this complex, no in vivo pharmacokinetic data are reported.

**Scheme 2:**
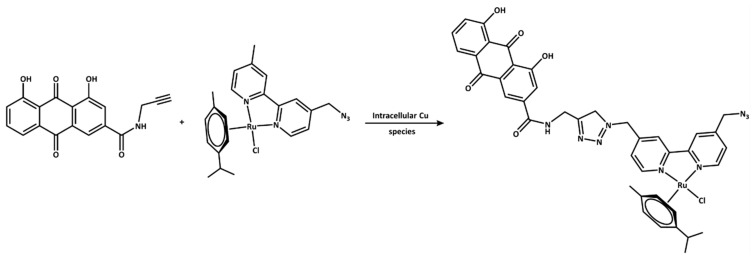
In situ generation of Ru-rhein complex via BCL strategy.

The remaining platinum and non-platinum entries of Table 2 are discussed in their respective metal subsections: the ER-stress/autophagy Pt(II) series [78]; the in situ bioorthogonally generated Ru-rhein complex (Section 4.2 and Scheme 2 [80]); the Complex I-directed gold(III) bisphosphine AuPhos-89 (Section 4.3 and Section 5.1 [81]); the liposomal Ir(III) construct shifting cell death toward ferroptosis/pyroptosis (Section 7 [82]); and the binuclear Re(I) tricarbonyl series (Figure 3A) acting through GSH disturbance (Section 4.3 [83]). For each, specific mitochondrial localisation evidence (ICP-MS fractionation or MitoTracker co-localisation) is stated at first mention.

OPT (pyriplatin-phosphonium ortho-isomer, Figure 3A) [79] represents the most mechanistically complete example of a mitochondria-targeted platinum complex within the reviewed period. ICP-MS fractionation shows its preferential accumulation in mitochondria over the nucleus, accompanied by multifactorial bioenergetic disruption, including reduced OXPHOS and glycolysis, cytochrome c release, and ultrastructural mitochondrial damage. Importantly, cytotoxicity is retained in cisplatin-resistant A549/Pt cells, consistent with its mechanistic independence from NER-mediated repair.

**Figure 4:**
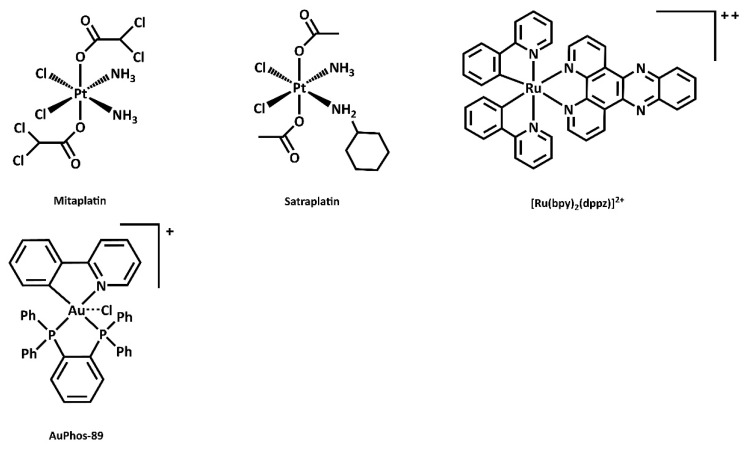
Other relevant metal complexes assessed in mitochondria targeting.

The Mitaplatin Pt(IV)/DCA (Figure 4) prodrug strategy, instead, exploits the concurrent pyruvate dehydrogenase kinase (PDK) inhibition to redirect pyruvate toward OXPHOS, creating a metabolic synthetic lethality in Warburg-phenotype cancer cells while achieving systemic stability through the kinetically inert Pt(IV) oxidation state [85].

### 4.2. Ruthenium Complexes

Ru(II) polypyridyl complexes combine ^3^MLCT-based phosphorescence (with ΦΔ, the quantum yield, up to 0.65), kinetic inertness of Ru(II)–N bonds, and inherent lipophilic cationic character—all without requiring TPP^+^ conjugation [86]. Cardiolipin-selective singlet oxygen (^1^O_2_) generation under irradiation has been documented for IMM-targeted dppz-Ru(II) complexes (Figure 4), which achieve IC_50_ values in the nanomolar range and therapeutic window ratios exceeding 500:1 in MCF-7 cells. This establishes a mechanistically specific photodynamic therapy (PDT) strategy. Singlet oxygen generated under photodynamic conditions induces oxidative modifications of cardiolipin, along with protein oxidation (e.g., cytochrome c), contributing to mitochondrial dysfunction and apoptosis. While cardiolipin is a primary and sensitive target, the process is not strictly selective and reflects a broader oxidative mechanism characteristic of PDT [87,88]. Another Ru(II) complex, but bearing a dppz derivative, uses its optimal hydrophilic–lipophilic balance upon activation to enter mitochondria, triggering: (i) oxidative stress (via antioxidant depletion and ROS generation), leading to mitochondrial dysfunction, DNA damage, and cell cycle arrest; (ii) apoptosis (via BAX/Bcl-2 regulation and caspase-9 activation); and mitophagy (via PINK1/Parkin upregulation), providing a dual mitochondrial attack [69]. In another study, the BCL (bioorthogonal catalysed lethality, Scheme 2) strategy is exploited, which is a targeted cancer therapy approach using bioorthogonal chemistry to generate active, toxic anticancer metallodrugs from non-toxic precursors exclusively within tumour cells, leaving healthy tissues unharmed. Thus, the active Ru-rhein (Figure 3) complex is generated in situ via endogenous copper in the tumour, thereby circumventing both plasma instability and thiol-mediated deactivation [80]—problems that commonly confound pre-formulated lipophilic cationic complexes.

### 4.3. Gold, Iridium, Osmium, Rhenium, and Vanadium Complexes

Au(I) and Au(III) complexes exploit TrxR2 selenylation (IC_50_ 30–80 nM for auranofin, Figure 3), with documented clinical target engagement [71,84]. Instead, cyclometallated Ir(III) complexes offer photophysically superior PLIM probes (ΦΔ up to 0.82; two-photon cross-sections > 200 GM at 800 nm) [65,66]. These enable sub-organelle oxygen mapping with mechanistic specificity, which is unattainable when using organic fluorophores. However, their kinetic inertness in vivo represents a translational barrier, which we address in Section 10. Os(II) polypyridyl complexes extend their theranostic utility into the NIR window (650–800 nm emission), relevant for in vivo optical imaging. Re(I) tricarbonyl complexes permit correlative FTIR/fluorescence tracking through their characteristic CO band signature, and binuclear Re(I) series have demonstrated in vivo tumour inhibition [89,90,91]. Finally, vanadyl porphyrin complexes partially preserve the PDT efficacy under hypoxia through type I photochemical pathways [92].

## 5. Mechanisms of Action

### 5.1. ETC Inhibition and Bioenergetic Disruption

Complex I (NADH-ubiquinone oxidoreductase) inhibition is the most documented ETC target among MTMCs from the mechanistic point of view. The best-supported example is AuPhos-89 (Figure 4) [81]: RNA-sequencing and quantitative proteomics converge on the perturbation of Complex I subunits as the primary network effect, with reduced OCR at sub-toxic concentrations and confirmed in vivo suppression of TNBC. Ru(II)-arene complexes have also been shown to inhibit Complex I, with inhibitory constants (Ki = 2.3 µM) comparable to those of rotenone (Ki = 1.8 µM), as determined by the Seahorse XF analysis [96,97,98]. The Seahorse Mito Stress Test measures key parameters of mitochondrial function by directly measuring the OCR of cells and has become the functional standard for mechanistic discrimination among uncoupling, substrate limitation, and direct ETC inhibition in MTMC studies. This assay uses sequential injection of oligomycin, FCCP, and rotenone/antimycin A to resolve OCR into its ATP-linked, maximal, and non-mitochondrial components [99].

### 5.2. Functional Assessment of Mitochondrial Integrity

Complementary to bioenergetic flux analysis, a small panel of fluorescent reporters is used to detect mitochondrial impairment at the single-cell level, each with characteristic strengths and limitations [100]. JC-1 reports membrane potential ratiometrically, forming red-emitting J-aggregates at high ΔΨm and reverting to green monomers upon depolarisation; the ratiometric readout is concentration-tolerant and well suited to flow cytometry, but the probe responds slowly and is prone to artefacts from uneven loading and aggregation. The single-wavelength rhodamine esters TMRM and TMRE respond rapidly and, when applied at low (“non-quench”) concentrations, permit calibrated, near-Nernstian estimation of ΔΨm. TMRM is generally preferred for quantitative work because TMRE binds membranes more avidly. MitoSOX and related mitochondria-targeted hydroethidine probes detect matrix superoxide, while boronate-based reporters detect hydrogen peroxide. All of them are sensitive but only semi-quantitative, and because they are themselves cationic and partition in a ΔΨm-dependent manner, they are confounded by the very potential changes they are used to interpret, mandating orthogonal confirmation and appropriate controls. Critically, every assay in this panel—like the oxygen-consumption-rate measurements above—reports a functional consequence of mitochondrial engagement, not the physical location of the test compound. Functional mitochondrial impairment does not necessarily demonstrate mitochondrial localization: a depolarising or ROS-inducing effect can arise from indirect, upstream, or downstream actions, and must be corroborated by the quantitative localization evidence defined in Section 6 before a complex is designated as mitochondria-targeting.

### 5.3. Redox Imbalance and Antioxidant System Disruption

Mitochondrial ROS overproduction by MTMCs arises through two complementary mechanisms: direct redox cycling, in which the metal complex is reduced at the ETC and transfers electrons to O_2_ as the terminal acceptor; and indirect ETC disruption, which prolongs the half-life of reduced flavin or ubisemiquinone intermediates that subsequently donate electrons to O_2_ [101,102]. Furthermore, GSH depletion occurs via metal-thiolate coordination, metal-catalysed oxidation, and TrxR inhibition (notably for gold complexes) [103,104,105]. The GLDH:ALT ratio, discussed further in the safety section, provides a clinical biomarker correlate for hepatic mitochondrial oxidative stress. A key *caveat* for translational studies is that the distinction between ROS as a pharmacological mechanism versus ROS as a driver of off-target toxicity in normal tissues cannot be reliably inferred from in vivo data alone.

### 5.4. Outer Mitochondrial Membrane Permeabilization and Multi-Modal Cell Death

MOMP is governed by the BAX/BAK versus BCL-2/BCL-XL/MCL-1 balance at the outer mitochondrial membrane. MTMCs engage this checkpoint through three distinct routes: cardiolipin peroxidation, direct perturbation of BCL-2 protein–protein interactions, and the collapse of ΔΨm [106,107,108]. A multi-modal cell death—defined as apoptosis co-occurring with ferroptosis, necroptosis, or autophagy—was observed in a substantial proportion of the reviewed studies. This complexity mandates an attribution using pharmacological rescue assays: z-VAD-fmk for apoptosis; ferrostatin-1 and GPx4 axis assessment for ferroptosis; necrostatin-1 and MLKL evaluation for necroptosis; and bafilomycin A1 or LC3-II flux for autophagy. Without such attribution, reported cytotoxicity mechanisms remain descriptive rather than causal [109,110,111].

### 5.5. MtDNA Damage and BER-Limited Repair

The 16.5 kb mitochondrial genome lacks histone protection and lies in close proximity to ETC-generated ROS, making it inherently susceptible to oxidative lesions such as 8-oxoguanine and strand breaks. Base excision repair (BER) is the primary—and likely the only complete—repair pathway for such damage [112]. Somatic mtDNA mutations occur in more than 80% of human cancers and are causally linked to ETC dysfunction and apoptotic resistance. MTMCs that accumulate in the matrix may produce mtDNA lesions at concentrations insufficient to damage nuclear DNA, which would provide a mechanistic basis for the selectivity claims made for mitaplatin-class agents. However, direct quantitative evidence for selective mtDNA damage over nuclear DNA damage in human-relevant models still remains limited [113,114,115].

## 6. Bioimaging and Theranostic Applications

### 6.1. Hierarchy of Localization Evidence

The inconsistent use of the term “mitochondria-targeting” across the metallodrug literature warrants explicit evidence standards, which we apply throughout this review. We distinguish three categories: (i) Mitochondria-targeting complexes [95,116,117,118] are those for which preferential mitochondrial accumulation is quantitatively demonstrated, either by subcellular fractionation with elemental quantification of the metal (ICP-MS measurement of the metal content in isolated mitochondrial versus cytosolic and nuclear fractions) or by quantitative co-localization microscopy reporting explicit Pearson correlation and/or Manders overlap coefficients against a validated mitochondrial counterstain (MitoTracker/MitoView); we adopt a working threshold of Pearson r ≥ 0.80 for unambiguous assignment. (ii) Mitochondria-localising complexes [93] are supported by qualitative imaging evidence of mitochondrial residence but lack quantitative accumulation data. (iii) Mitochondrion-perturbing complexes alter mitochondrial function (ΔΨm collapse, ROS generation, respiratory inhibition) without any direct localization evidence. Only categories (i) and (ii) are treated as mitochondria-targeting. Category (iii) agents, including the antimetastatic complex NAMI-A, are discussed as mechanistic comparators. The justification is methodological: functional readouts measure a downstream consequence and cannot localise the analyte, whereas fractionation ICP-MS and quantitative co-localization measure the metal itself at its purported site of action. Robust category-(i) exemplars now exist across the periodic table—the monofunctional platinum complex OPT, shown by subcellular ICP-MS fractionation to accumulate predominantly in mitochondria [79]; cyclometalated Ir(III) complexes (**1**, Figure 3A) combining Pearson coefficients of 0.74–0.87 with confirmatory ICP-MS fractionation [93] and a viscosity-sensing Ir(III) complex (**2**, Figure 3A) reaching Pearson 0.93 [94]; [Os(btzpy)_2_](PF6)_2_ (btzpy = 2,6-bis(1-phenyl-1,2,3-triazol-4-yl)pyridine), an Os(II) triazole complex (Pearson 0.85) (Figure 3A) [95]; and Re(I) tricarbonyl complexes (**3**, Figure 3A) attaining Pearson 0.918 [83]—establishing that the standard proposed here is readily attainable with current methods.

### 6.2. Cellular Demonstration of Mitochondrial Localisation

For luminescent Ru(II) polypyridyl and cyclometalated Ir(III) complexes, mitochondrial residence is most often established by confocal co-localisation with MitoTracker counterstains, quantified by Pearson and Manders’ coefficients; their long-lived ^3^MLCT phosphorescence permits time-gated imaging that rejects nanosecond autofluorescence. This optical evidence—rather than ICP-MS fractionation alone—constitutes the dominant localisation proof for these metal series and is the basis on which they satisfy the inclusion criteria defined in Section 2.2.

FLIM (fluorescence lifetime imaging microscopy) and PLIM (phosphorescence lifetime imaging microscopy) exploit the microsecond-domain phosphorescence lifetimes of Ru(II) and Ir(III) complexes (0.1–10 µs) to achieve concentration-independent mitochondrial imaging with a complete rejection of nanosecond autofluorescence [119]. Intramitochondrial oxygen mapping by PLIM, which relies on Stern–Volmer quenching kinetics, has resolved oxygen concentrations of 5–20 µM in living cancer cells—substantially below bulk extracellular values [120]. This resolution is sufficient to characterise a metabolic heterogeneity in three-dimensional tumour spheroids.

^99^^m^Tc-MIBI—a ΔΨm-sensitive lipophilic radiotracer—serves both as a clinical imaging agent and as a pharmacological model for MTMC accumulation dynamics. Its MDR1/P-gp sensitivity directly informs the interpretation of in vivo accumulation data for TPP^+^-conjugated candidates [121]. Finally, ^68^Ga-TPP PET [122] represents a next-generation voltage-sensing tracer, offering superior spatial resolution for preclinical stratification.

## 7. Nanotechnology and Delivery Systems

Metal–organic frameworks with porphyrin linkers (PS-MOFs; Hf^4+^ or Zr^4+^ nodes) [123] eliminate the porphyrin self-quenching through spatial isolation, substantially enhancing ^1^O_2_ quantum yield. Lin et al. demonstrated that TPP^+^-lipid-modified Hf-porphyrin MOFs achieve co-localization coefficients of 0.91 with MitoTracker and produce 87% tumour growth inhibition in CT26 syngeneic models at sub-lethal doses [124,125]. The syngeneic system preserves immune competence, which may contribute to efficacy through cGAS-STING pathway activation triggered by mtDNA-release-dependent immunogenic cell death. The liposomal encapsulation of Ir(III) complexes shifts the predominant cell death mechanism from apoptosis-dominant to ferroptosis and pyroptosis, suggesting that the delivery context modulates the pharmacodynamic programme via differences in intracellular concentration kinetics [82]. Polymeric nanocarriers and Szeto-Schiller peptide surface modification strategies provide additional mitochondrial targeting layers superimposed on passive tumour accumulation based on enhanced permeability and the retention (EPR) effect [126,127]. However, EPR dependence itself represents a clinical limitation in tumours lacking adequate vascular permeability [128].

## 8. Analytical Approach and Clinical Pharmacokinetic Interpretation

### 8.1. Total Metal Quantification Versus Active Drug Species

A recurrent conceptual and methodological limitation in metallodrug pharmacokinetics is the implicit assumption that total metal concentration directly reflects exposure to pharmacologically active drug species [129]. When biological samples containing metallodrugs are analysed by inductively coupled plasma–mass spectrometry (ICP-MS) without prior chromatographic separation [130,131,132], the technique quantifies only the total element content and does not distinguish among chemically distinct metal-containing forms. Consequently, the detected signal may represent the combined contribution of multiple species with substantially different pharmacological, toxicological, and kinetic properties, including the following:

(i) Intact parent complexes, retaining their original coordination sphere and pharmacological activity;
(ii) Aquated or ligand-exchange products formed by reactions with water, chloride, or endogenous nucleophiles;
(iii) High-molecular-weight protein-associated fraction, coordinatively saturated adducts with plasma proteins—principally albumin and transferrin—which are typically inactive and non-releasable under physiological conditions;
(iv) Low-molecular-weight thiol adducts (glutathione, cysteine, methionine);
(v) Hydrolytic, redox-derived, or partially degraded metal-containing products [133].

Following IUPAC terminology, the separation of metal-containing forms by size or charge constitutes fractionation, whereas speciation analysis requires identification of defined chemical species; size-exclusion and capillary-electrophoresis ICP-MS therefore perform species-selective fractionation rather than full speciation unless coupled to structural identification. The quantitative subcellular distribution is further constrained by the fractionation fidelity: even under optimised protocols, only ~18% and ~66% of cellular platinum are recovered in nuclear and cytosolic fractions (≈ 81% overall), and recovery degrades with fraction impurity and accumulated uncertainty [134]; lysis can itself alter speciation. Reported mitochondrial accumulation values should therefore be interpreted as operational estimates, not as absolute partition coefficients.

Since these species differ fundamentally in bioavailability, target-binding affinity, and toxicological profile, the total element concentration cannot automatically be interpreted as equivalent to exposure to pharmacologically relevant drug species. The consequences affect dose-finding, exposure-response modelling and drug–drug interaction assessments. However, total elemental measurements and pharmacologically meaningful exposure represent related but fundamentally distinct concepts. This difference becomes particularly relevant for metallodrugs undergoing extensive irreversible protein association.

Kato et al. [135] demonstrated this directly in rats: the terminal elimination half-life of intact cisplatin, measured by UPLC-MS/MS, was approximately 40 min, whereas total platinum, measured by ICP-MS, exhibited a terminal half-life exceeding five days—a discrepancy of more than 180-fold and attributable largely to persistent albumin adduction. In human patients, total plasma Pt clearance is approximately 0.68 L/h, while ultrafilterable Pt clearance is approximately 35.5 L/h, a 52-fold difference [136,137]. Oxaliplatin similarly reaches 85% irreversible protein binding within 24 h [138,139]. By contrast, carboplatin exhibits substantially lower protein binding (15–40%, with approximately only 10% irreversible binding), making total Pt a more reasonable surrogate marker of systemic exposure and supporting the clinically validated Calvert formula-based therapeutic drug monitoring strategy used for individualised carboplatin dosing in oncology, determining the total dose (mg) needed to achieve a target area under the curve (AUC) based on renal function [129,140].

Techniques such as CE-ICP-MS or SEC-ICP-MS identify the distribution of metals across electrophoretic or molecular-weight fractions rather than definitively characterising molecular structures or coordination environments. For KP1019 (BOLD-100), CE-ICP-MS indicates that 98–99% of ruthenium, quantified in published Phase I plasma studies, likely reflects protein-associated species rather than intact parent complexes [141]. Similarly, for auranofin, the intact parent compound is essentially undetectable in blood, whereas the plasma gold half-life (∼26 days) predominantly reflects persistent protein-associated Au-metabolite [142].

### 8.2. Analytical Hierarchy: From Total Metal to Species-Specific Quantification

Rather than benchmarking individual instruments—which is peripheral to the aims of this review—it is more useful to recognise three conceptual analytical tiers, summarised in Table 3. Total-element ICP-MS is a rapid and clinical routine but provides no species discrimination; size- or charge-based hyphenated approaches (SEC-ICP-MS, CE-ICP-MS) perform a species-selective fractionation that separates protein-bound from free metal without resolving the molecular structure; and species-specific UHPLC-ICP-MS quantifies the intact parent complex separately from its metabolites and adducts and has been validated for clinical use (oxaliplatin) [133,143,144,145]. The practical implication for the metallodrug pharmacokinetics is the one developed in Section 8.1: only the species-specific tier reports pharmacologically meaningful exposure, whereas total metal and fractionation data must be interpreted with the speciation caveats noted above.

### 8.3. The Regulatory Gap and Clinical Laboratory Feasibility

No specific FDA or EMA guidance document currently mandates metal speciation as a required component of metallodrug clinical pharmacokinetic (PK) submissions [144]. The current regulatory practice accepts total platinum measured by AAS or ICP-MS as the primary pharmacokinetic analyte for cisplatin and carboplatin submissions—a convention that predates the availability of validated hyphenated techniques [144]. The EMA Bioanalytical Method Validation Guideline (EMEA/CHMP/EWP/192217/2009 Rev. 1) [151] provides a general framework applicable to ICP-MS methods, but does not specify speciation as a required analytical dimension. In hospital laboratories with pre-existing ICP-MS infrastructure, UHPLC-ICP-MS for intact drug speciation is technically implementable without major capital expenditure beyond the UHPLC system [152]. The primary barriers are method validation, availability of reference materials, and the absence of regulatory incentives. Overcoming this regulatory inertia is a field-level priority. For next-generation MTMC development, speciation-aware PK is not just analytically desirable, but also mechanistically essential: a complex whose activity depends on an intact lipophilic cationic scaffold accumulating in mitochondria via ΔΨm cannot have its target-site exposure reliably inferred from total metal measurements in plasma [153].

## 9. Clinical Safety Monitoring Framework for Mitochondria-Targeting Metallodrugs

Since MTMCs disrupt oxidative phosphorylation and engage redox-dependent death pathways (*vide supra*), early-phase safety monitoring should prioritise tissues with high oxidative phosphorylation dependence (myocardium, renal proximal tubule, hepatocytes, and dorsal root ganglion neurons). Among available biomarkers, glutamate dehydrogenase (GLDH) and the GLDH:ALT ratio are mechanistically the most informative [154,155,156], being specific to mitochondrial-matrix injury, while plasma lactate provides a systemic surrogate of oxidative phosphorylation failure. A comprehensive organ-by-organ monitoring framework—integrating cardiac (high-sensitivity troponin and global longitudinal strain), renal (KIM-1 and cystatin C) and neurological (FACT/GOG-NTX and neurofilament light chain) endpoints—exceeds the scope of the present review, given that few MTMCs have reached in vivo testing, and will therefore be developed in a dedicated companion article. The principle to be retained here is that safety surveillance for mitochondria-targeting agents must be grounded in organelle-level pathophysiology rather than transplanted from DNA-damaging platinum templates.

A critical appraisal of the MTMC literature that confines itself to mechanistically sophisticated in vitro findings—without systematic analysis of compounds that have failed in clinical development—risks perpetuating the field’s historically poor translation record. Of an estimated several thousand transition metal complexes with documented in vitro anticancer activity published between 2000 and 2026, fewer than 30 have entered clinical trials, and only three have achieved regulatory approval. The compounds summarised in Table 4 are instructive not because they represent idiosyncratic failures, but because they embody recurring pharmacological and analytical patterns that must be explicitly addressed in any next-generation MTMC programme.

The in vitro selectivity index does not reliably predict the in vivo therapeutic index. The translation gap in metallodrug oncology is not primarily a synthesis issue, but an analytical, pharmacokinetic and safety biology problem.

## 10. Case Studies in Clinical Translational Failure

KP1019 (Figure 3B) and its sodium salt NKP-1339/BOLD-100 represent the most thoroughly documented trajectory from a mechanistic promise to a formulation-limited clinical constraint. KP1019 achieved disease stabilisation in five of six evaluable Phase I patients but was halted by aqueous solubility of approximately 600 µM—insufficient to reach therapeutically relevant concentrations in feasible infusion volumes. Its more soluble sodium salt, BOLD-100, has advanced to Phase I (MTD 625 mg/m^2^; DCR 26% in 38 evaluable patients [148]) and Phase 1b/2a in conjunction with the combination chemotherapy regimen FOLFOX (NCT04421820; Grade 3/4 neutropenia 42%). Neither compound possesses a validated predictive biomarker for patient selection, limiting precision medicine positioning [157,158].

NAMI-A (Figure 3B) (IC_50_ > 300 µM), on the other hand, has an antimetastatic rather than cytotoxic primary mechanism. It produced only one partial response in 27 evaluable Phase I/II patients treated with gemcitabine and was declared insufficiently effective. This illustrates that murine syngeneic antimetastatic activity is not a reliable efficacy surrogate for human clinical benefit [159].

Titanocene dichloride (Figure 3B) (IC_50_ ∼2000 µM; requires an acidic malate buffer at pH 3.2–3.5 with rapid TiO_2_ formation at physiological pH) produced zero objective responses in Phase II despite promising xenograft data. This demonstrates that efficacy data derived from a chemically unstable species have no translational validity [160,161].

Budotitane (Figure 3B) was discontinued after Phase I because of cardiac arrhythmia attributed partly to the Cremophor EL formulation. Its inseparable cis-α/cis-β isomers prevented pharmacokinetic attribution to a defined chemical entity [162].

Satraplatin (Figure 4) (Phase III SPARC trial, n = 950) met its progression-free survival endpoint (HR = 0.67; *p* < 0.001), but not overall survival, and the FDA denied approval in 2007. At least six plasma biotransformation products were identified with an undetectable parent compound, making the dose–response modelling pharmacologically uninterpretable [163].

RAPTA-C (Figure 3B) has never entered clinical development due to its IC_50_ > 100 µM, an antimetastatic mechanism not captured by standard xenograft endpoints, and daily dosing requirements that are incompatible with intravenous administration schedules [164].

### 10.1. Recurrent Mechanisms of Translational Failure

Analysis of these compounds, together with the broader MTMC attrition record, identifies six recurring mechanistic categories of translational failure that collectively account for the majority of clinical programme discontinuations.

Poor aqueous solubility imposes infusion volume ceilings that hold plasma concentrations at a sub-therapeutic level (KP1019, RAPTA-C, and several Ir(III) theranostics). Rapid hydrolysis or ligand loss at a physiological pH generates inert metal-oxide species that are incompatible with target engagement, as is the case for titanocene and budotitane. Extensive plasma-protein binding reduces the pharmacologically active free fraction to below 2–5% of total metal—≈98–99% of ruthenium is albumin-bound for KP1019, and more than 95% of platinum is bound for cisplatin by 24 h. Non-specific biodistribution, with predominant accumulation in the hepatic, splenic, and renal reticuloendothelial system, competes with tumour delivery and drives off-target toxicity. Kinetic inertness can prevent in vivo target engagement, a barrier that is particularly relevant for Ir(III) complexes whose thermodynamic stability constrains metal–ligand dissociation. Finally, metabolic inactivation or reductive decomposition—exemplified by a reduction of Au(III) to Au(I) or Au(0) in the intracellular reducing milieu—abolishes the intended active species before it reaches its target.

### 10.2. Quantitative Reassessment of the ΔΨm Selectivity Paradigm

The electrophoretic selectivity model—which predicts preferential mitochondrial MTMC accumulation in cancer cells supposedly having a hyperpolarised ΔΨm—requires critical reassessment in light of calibrated intact-cell measurements. Using TMRM-based ratiometric calibration, Rovini et al. measured intact-cell ΔΨm values of −131.33 ± 10.37 mV in HepG2 cells and −131.68 ± 2.47 mV in BJ1 fibroblasts [50]. These values are statistically indistinguishable, directly contradicting the assumption of systematic cancer-cell hyperpolarisation. The much larger values frequently cited as evidence of tumour hyperpolarisation (−180 to −220 mV) derive from isolated mitochondria in resting State 4, which are not comparable to intact-cell measurements that integrate plasma membrane potential, cytosolic buffering, and probe equilibration kinetics.

The practical consequence is that the in vitro selectivity index (SI = IC_50_ normal/IC_50_ cancer), measured under standard 2D culture conditions without protein binding, metabolic transformation, or microenvironmental complexity, does not predict the in vivo therapeutic index. This is illustrated by two examples: titanocene dichloride, which showed an apparently favourable SI in vitro yet produced zero responses in Phase II; and NAMI-A, which exhibited antimetastatic activity in murine models, but no clinical antitumour activity. Meanwhile, tissues with the highest OXPHOS dependence—cardiomyocytes (30–40% mitochondrial volume fraction), proximal tubular cells, hepatocytes, and dorsal root ganglion neurons—remain at risk of off-target MTMC accumulation regardless of the in vivo SI.

Resistance to MTMCs is mechanistically distinct from the NER- and MMR-based resistance that limits conventional platinum drugs. ΔΨm reduction—whether through uncoupling protein (UCP) upregulation, ETC stoichiometry remodelling, or adaptive ATP synthase modification—would abolish the electrophoretic driving force for lipophilic cation accumulation. This constitutes the most MTMC-specific resistance axis, although systematic experimental characterisation in drug-selected resistant models remains limited. MDR1/P-gp efflux of lipophilic substrates, documented for ^99^^m^Tc-MIBI [121], is directly relevant to TPP^+^-conjugated MTMC candidates. This suggests that MDR1 functional assessment should be incorporated into resistance profiling. NRF2/ARE antioxidant upregulation—arising from constitutive KEAP1 mutation or methylation—elevates the ROS threshold above the pharmacological ceiling of ROS-generating MTMCs. BSO (a glutamate-cysteine ligase inhibitor) and NRF2/KEAP1 protein–protein interaction inhibitors represent rational combination partners. BCL-2 overexpression, although shared with cisplatin resistance, may be partially overcome by MTMCs that generate ROS of sufficient amplitude to exceed BCL-2 sequestration capacity, as suggested by the retained activity in BCL-2-overexpressing lines for some gold and iridium complexes. Finally, protective mitophagy—PINK1/Parkin-mediated removal of damaged mitochondria before death commitment—represents an emerging MTMC-specific resistance mechanism that may be addressed by mdvi-1 or liensinine-based combination strategies [165,166,167,168].

## 11. Critical Appraisal and Future Directions

### 11.1. Key Limitations of the Current Evidence Base

Three structural limitations constrain the translational interpretability of the MTMC literature from 2016 to 2026. First, cell line representativeness is insufficient. The majority of mechanistic studies employ MCF-7, A549, HeLa, and HepG2 lines, which may not represent the tumour types and mitochondrial phenotypes for which MTMC strategies are most clinically relevant. Expansion to patient-derived organoids (PDOs), syngeneic immunocompetent systems, and cell panels stratified by mitochondrial phenotype (ΔΨm, OXPHOS dependency, NRF2 status, and BCL-2 expression) is necessary before pharmacological conclusions can be generalised. Second, the intracellular speciation is chronically undercharacterised. The predominant approach—ICP-MS quantification of total metal in subcellular fractions—provides no information on the chemical identity of metal-containing species. It conflates intact complexes, aquation products, GSH adducts, protein-bound metal and free metal ions into a single measurement. Adoption of LC-ICP-MS, CE-ICP-MS, and native metalloproteomic approaches as standard characterisation elements is a methodological priority.

Third, in vivo pharmacokinetic characterisation is available for only a minority of reported complexes. In its absence, dose-efficacy relationships and projected human dosing regimens lack a quantitative foundation [169,170,171,172,173].

### 11.2. Emerging Directions

Graph neural networks (GNNs) trained on existing structure–activity relationship datasets for coordination chemistry represent a tractable, ML-assisted design framework for the relatively compact chemical space of metal complexes. Input features would include ligand topology, metal centre identity, predicted reduction potential, and experimental lipophilicity, with cytotoxicity and mitochondrial targeting efficiency as the outcome variables.

Multi-target metallodrug design may provide pharmacological redundancy against adaptation through single resistance mechanisms. Examples include combining ETC Complex I inhibition with TrxR inhibition, pairing PDT sensitisation with protein adduct formation, or combining mtDNA binding with antioxidant enzyme inhibition.

Combination therapy is mechanistically supported for the use of several classes of agents: BCL-2 inhibitors (venetoclax, BH3 mimetics), immune checkpoint inhibitors that exploit cGAS-STING pathway activation triggered by mtDNA release, and NRF2 inhibitors. Such combinations warrant a systematic preclinical evaluation using defined synergy metrics, such as Chou–Talalay combination index analysis for determining drug interactions.

Biomarker-guided clinical trial design represents the most conceptually coherent approach to precision medicine positioning of MTMC candidates. This would involve pre-treatment ΔΨm imaging—using ^99^^m^Tc-MIBI SPECT or ^68^Ga-TPP PET—for patient stratification and selecting individuals with functionally demonstrated mitochondrial hyperpolarisation and low MDR1 expression. Prospective validation of this approach is pending [174,175,176,177].

## 12. Conclusions

The MTMC literature from 2016 to 2026 documents a mechanistically sophisticated evolution, from nuclear DNA-centric to mitochondria-targeted metallopharmacology. Across seven metal series (Pt(II/IV), Ru(II/III), Au(I/III), Ir(III), Os(II), Re(I), and V(IV/V)), the field has converged on four primary intramitochondrial target categories: disruption of the ETC and OXPHOS, perturbation of redox homeostasis, MOMP-dependent apoptotic gating, and compromise of mtDNA integrity. The observation of multi-modal cell death across a substantial proportion of reviewed studies reflects the mitochondrion’s role as a hub for multiple programmed death pathways.

Clinical proof-of-concept for mitochondrial target engagement already exists. TrxR2 inhibition has been confirmed ex vivo in chronic lymphocytic leukaemia (CLL) patients receiving auranofin, and manageable tolerability has been established for BOLD-100 in platinum-resistant solid tumours. These findings validate the pharmacological premise. Nevertheless, the clinical attrition rate for non-platinum metallodrugs, as a class, exceeds 99.9%, indicating that the translational framework must be rebuilt.

Three corrections to prevailing practice are necessary for substantive translational progress, as follows:

1. Adoption of speciation-aware pharmacokinetics using hyphenated ICP-MS techniques rather than total metal measurement.
2. Structured, organ-specific clinical safety monitoring that incorporates mitochondria-specific biomarkers (GLDH, GLDH:ALT ratio, plasma lactate, KIM-1, and hs-cTnI/T, GLS) instead of transplanted cisplatin safety templates.
3. A critical re-evaluation of in vitro selectivity indices as predictors of therapeutic indices, given the demonstrated equivalence of intact-cell ΔΨm between matched cancer and normal cell pairs and the established in vivo failure of compounds with an apparently favourable in vitro SI.

These corrections require two parallel actions. First, regulatory engagement, particularly the development of metal speciation-aware PK guidance within existing EMA and FDA bioanalytical frameworks. Second, community investment in patient-derived preclinical models and biomarker-stratified clinical trial designs.

The bioinorganic chemistry community now possesses convergent tools: AI-assisted multi-target design, integrative metalloproteomics, and voltage-sensitive PET theranostics. These position the field to deliver the next generation of mitochondria-targeted precision metallopharmaceuticals. Realising this potential, however, requires that the field’s analytical and translational infrastructure be brought into alignment with its mechanistic ambitions.

The decade of work surveyed here establishes that mitochondria-targeting metal complexes are mechanistically diverse and, in the best-characterised cases, genuinely organelle selective; yet it also exposes a conceptual weakness that now limits the field more than any synthetic barrier does. A large body of literature designates complexes as “mitochondria-targeting” on the strength of functional perturbation alone—a fall in membrane potential, a rise in reactive oxygen species, or a change in respiration—without ever demonstrating that the complex reaches the organelle. The central message of this review is therefore a cautionary one: mitochondrial dysfunction alone should not be considered evidence of mitochondrial accumulation. Functional readouts measure consequences that can arise from indirect or downstream actions, whereas the claim of targeting is a statement about location and must be supported by evidence that measures the metal at its site of action. We have accordingly proposed a localization evidence hierarchy in which the designation “targeting” is reserved for complexes with quantitative subcellular ICP-MS fractionation or co-localization data bearing explicit Pearson/Manders coefficients, and we have shown that this standard is readily achievable with established methods.

A second recalibration concerns disposition. The thermodynamic, ΔΨm-driven model remains a valuable design heuristic, but it is an idealised equilibrium description: the real intracellular distribution reflects membrane partitioning, protein binding, pH gradient, kinetic limits, and intracellular sequestration, and cancer-cell membrane potential is heterogeneous rather than uniformly elevated. Equally, since inductively coupled plasma–mass spectrometry quantifies the total element and cannot distinguish intact complex from protein adducts, aquation products, and decomposition species, speciation-aware pharmacokinetics is not an optional refinement but a prerequisite for interpreting exposure, attributing toxicity, and rationalising dose. The persistent translational barriers—poor aqueous solubility, rapid ligand exchange, extensive irreversible protein binding, and non-specific biodistribution—are precisely those that total metal pharmacokinetics renders invisible.

The most credible path to clinical translation thus runs through the analytical and pharmacokinetic discipline rather than through novel chemotypes alone: routine speciation analysis, prospective and quantitative localization evidence, organelle-level safety biomarkers, and biomarker-guided patient stratification. Metal complexes that satisfy these criteria—demonstrably mitochondria-targeting, speciation-characterised, and pharmacokinetically defined—represent the rational basis for the next phase of mitochondria-directed precision oncology, and the framework advanced here is intended to make the comparison among candidate complexes both rigorous and reproducible.

## Figures and Tables

**Table 1:** Summary of the most common ligands used in MTMCs and their main characteristics.

Metal	Common Ligands/Strategies	Key Advantages and Mechanisms	Refs.
Ir	Cyclometalated complexes	Excellent photophysical properties (can be used for imaging and therapy), ability to induce immunogenic cell death by disrupting metal ion homeostasis, high tunability of ligand structure for precise targeting.	[65,66,67]
Ru	Half-sandwich complexes, dppz-based compounds	Highly effective at inducing oxidative stress and apoptosis, superior cytotoxicity to cisplatin in many models, triggers both apoptosis and mitophagy, can disrupt key metabolic pathways (OXPHOS, TCA cycle).	[68,69,70]
Re	dppz-based complexes	Effective against aggressive cancers like triple-negative breast cancer and disrupts metal ion homeostasis, leading to protein degradation and activating the immune system against the tumour.	[69]
Au	N-heterocyclic carbene (NHC) pincer ligands	Promotes mitochondria-mediated cell death by increasing ROS production.	[71]
Zn, Cu, Pt	Thiosemicarbazone complexes, NHC pincer ligands	Cu and Zn complexes are common with thiosemicarbazone ligands; Pt complexes can also be designed to target mitochondria rather than DNA.	[72,73]

**Table 2:** Representative MTMCs (2016–2026). TNBC = triple-negative breast cancer; GSDMD = gasdermin D; CLL = chronic lymphocytic leukaemia. Note: _nn_^m^Tc-MIBI and ^68^Ga-TPP are ΔΨm-/efflux-sensitive diagnostic radiotracers, not therapeutic agents; they are discussed only as accumulation proxies (Section 6) and are excluded from this therapeutic comparison.

Compound	Ligand/Scaffold	Mito Targeting Strategy	Primary Mito Mechanism	Model	Stage	Key PK Data	Ref.
PIP-platin Pt(II)	Cyclometallated cationic	Intrinsic cationic; ICP-MS confirmed	ROS ↑, ΔΨm ↓, CytC release, necrosis; β-catenin trafficking	Multi-line in vitro; migration assays	Preclinical	Trafficking characterised; no PK data	[77]
Pt(II)-mito (ER-stress/autophagy)	Mito-targeted series	Declared mito targeting	Apoptosis + autophagic death via ER-stress	In vitro A549	Preclinical	No PK data reported.	[78]
OPT (pyriplatin-TPP) Pt(II)	Monofunctional + –CH_2_Ph_3_P^+^	TPP^+^ delocalized cation; mito > nucleus (ICP-MS)	OXPHOS + glycolysis disruption; CytC release; ultrastructural mito damage	In vitro + in vivo (NSCLC nude mice)	Preclinical advanced	Biodistribution + uptake characterised; cisplatin comparison	[79]
Ru-rhein (BCL, in situ) Ru(II)	Ru-N_3_ + rhein-alkyne → CuAAC	Bioorthogonal in situ synthesis; endogenous Cu	ROS ↑, mito damage, autophagy	In vitro + in vivo (xenograft A549)	Preclinical advanced	Anti-instability design; systemic inactivation avoided	[80]
AuPhos-89 Au(III)	Bis-phosphine	ETC-directed; Complex I primary target	ETC Complex I inh.; ATP ↓; RNA-seq + proteomics	In vitro + in vivo (TNBC)	Preclinical advanced	Physiological stability emphasised; multi-omics support	[81]
Ir(III)-mito liposome	Ir(III) + liposomal nanodelivery	Mito targeting + nanodelivery PK strategy	Ferroptosis + gasdermin D pyroptosis (GSDMD)	Preclinical in vitro	Preclinical	Nanodelivery as PK/safety strategy	[82]
ReN/ReS (binuclear Re(I))	[Re_2_(CO)_6_(dip)_2_L]^2+^	Mito-accumulating cationic	ROS ↑; GSH disturbance; apoptosis + necroptosis	In vitro + in vivo (xenograft)	Preclinical advanced	GSH reactivity documented; thiol sink	[83]
Auranofin Au(I)	Phosphine–Au(I)–thiosugar	TrxR2 + TrxR1 inhibition; SeCys selenylation	TrxR2 IC_50_ 30–80 nM; redox collapse; MOMP	In vitro + in vivo + Phase II CLL	Phase II (7/13 responders; GI/ocular tox)	TrxR2 inhibition confirmed ex vivo PBMCs; t½ ~26 days (Au metabolites)	[84]

**Table 3:** Analytical platforms for metallodrug species-selective fractionation and speciation analysis. LOD = limit of detection; LOQ = limit of quantification; CV = coefficient of variation; IDMS = isotope dilution mass spectrometry; TDM = therapeutic drug monitoring; MW = molecular weight.

Technique	What It Measures	Strengths	Limitations	Clinical Readiness	Ref.
ICP-MS (standalone)	Total elemental metal; no species discrimination	Rapid (2–5 min); sub-ppb LOD; routine clinical lab; standard for total Pt TDM	Cannot distinguish intact drug, metabolites, protein adducts, inactive species	Routine clinical	[145,146]
SEC-ICP-MS	Protein-bound (>10 kDa) vs. free fractions by molecular weight	Non-denaturing; femtomolar LOD for Pt; preserves native adduct stoichiometry	20–40 min run; does not resolve thiol adducts within same MW window	Research; near-clinical	[145,147]
UHPLC-ICP-MS (intact drug)	Intact parent drug separated from metabolites and adducts	LOQ 50 nM; accuracy 96.8–103%; precision < 2.5% CV; stability 473 days at −80 °C; validated clinically for oxaliplatin	Species-specific validation per compound; metabolite ID requires orthogonal MS	Validated for clinical use (oxaliplatin)	[145,148,149]
HPLC-ICP-IDMS	Absolute quantification of all metal-containing species without species-specific standards via post-column isotope dilution	Species-independent absolute quantification; applicable to unknowns; no reference standard needed for metabolites	Instrument cost ~550K–1M $; no commercial isotope-labelled spikes for most MTMCs; expertise-intensive	Reference laboratories only	[145,150]

**Table 4:** Representative translational failures of metal-based anticancer agents. IC_50_ values from standard 2D cell culture assays unless specified. IND = Investigational New Drug application; DLT = dose-limiting toxicity; OS = overall survival; PFS = progression-free survival; mRCC = metastatic renal cell carcinoma; PR = partial response; SD = stable disease; PK/PD = pharmacokinetic/pharmacodynamic linkage.

Compound	Metal	Clinical Phase	Primary Limitation	Outcome and Key Lesson	Refs.
KP1019	Ru(III)	Phase I (completed)	Aqueous solubility ~600 µM; infusion volume ceiling; ≥98% Ru albumin-bound	Disease stabilisation in 5/6 patients; halted by formulation, not toxicity. Replaced by NKP-1339/BOLD-100. Lesson: speciation and solubility must precede Phase I entry.	[141,157,158]
NAMI-A	Ru(III)	Phase I/II + gemcitabine	IC50 > 300 µM; antimetastatic not cytotoxic; murine syngeneic efficacy did not translate	1 PR, 10 SD in 27 evaluable patients; declared insufficiently effective. Lesson: murine antimetastatic endpoints are not reliable surrogates for human clinical benefit.	[159]
Titanocene dichloride	Ti(IV)	Phase II (completed)	IC50 ~2000 µM; rapid TiO2 formation at pH > 3.5; requires acidic malate buffer (pH 3.2–3.5)	Zero objective responses in mRCC and metastatic breast cancer. Lesson: speciation stability at physiological pH must be validated before efficacy extrapolation.	[160,161]
Budotitane	Ti(IV)	Phase I (discontinued)	Cardiac arrhythmia DLT (Cremophor EL formulation); inseparable cis-α/cis-β isomers preventing PK attribution	Development halted by DLT. Lesson: chemical purity, defined speciation, and formulation biocompatibility are prerequisites for clinical entry.	[162]
Satraplatin	Pt(IV)	Phase III SPARC (n = 950)	No OS benefit (PFS HR 0.67 met; FDA denied 2007); ≥6 plasma metabolites; parent undetectable in plasma	Phase III failure at primary survival endpoint; myelosuppression DLT. Lesson: Phase III endpoints and PK/PD linkage must be established before pivotal trial.	[163]
RAPTA-C	Ru(II)-arene	Preclinical (no IND)	IC50 > 100 µM; antimetastatic mechanism not captured by xenograft endpoints; solubility constraints; daily dosing required	Has not entered IND-enabling studies. Lesson: mechanisms must align with regulatory and clinical endpoint framework for chosen indication.	[164]

## Data Availability

The original contributions presented in this study are included in the article. Further inquiries can be directed to the corresponding author(s).

## Abbreviations

The following abbreviations are used in this manuscript:

AAS	atomic absorption spectrometry
ACN	acetonitrile
AKI	acute kidney injury
ALP	alkaline phosphatase
ALT	alanine transaminase
ARE	antioxidant response element
AST	aspartate transaminase
ATP	adenosine triphosphate
AUC	area under the curve
BAK	BCL-2 homologous antagonist-killer
BAX	BCL-2-associated X protein
BCL	bioorthogonally catalysed lethality
BCL-2	B-cell lymphoma 2
BCL-XL	B-cell lymphoma-extra large
BER	base excision repair
BNP	B-type natriuretic peptide
bpy	2,2′-bipyridine
BSO	buthionine sulfoximine
CE-ICP-MS	capillary electrophoresis–inductively coupled plasma mass spectrometry
cGAS-STING	cyclic GMP-AMP synthase–stimulator of interferon genes
CIPN	chemotherapy-induced peripheral neuropathy
CKD-EPI	Chronic Kidney Disease Epidemiology Collaboration (formula)
CLL	chronic lymphocytic leukaemia
CTCAE	Common Terminology Criteria for Adverse Events
CTR1	high-affinity copper transporter 1
CTRCD	cancer therapy-related cardiac dysfunction
CuAAC	copper-catalysed azide-alkyne cycloaddition
CV	coefficient of variation
DCR	disease control rate
DILI	drug-induced liver injury
DLT	dose-limiting toxicity
dppz	dipyrido[3,2-a:2′,3′-c]phenazine
ECG	electrocardiogram
eGFR	estimated glomerular filtration rate
EMA	European Medicines Agency
EOT	end of treatment
EPR	enhanced permeability and retention
ERCC1	excision repair cross-complementation group 1
ETC	electron transport chain
FACT/GOG-NTX	Functional Assessment of Cancer Therapy/Gynecologic Oncology Group-Neurotoxicity
FCCP	carbonyl cyanide-4-(trifluoromethoxy)phenylhydrazone
FDA	Food and Drug Administration
FLIM	fluorescence lifetime imaging microscopy
FOLFOX	folinic acid, fluorouracil, and oxaliplatin (combination chemotherapy regimen)
F/U	follow-up
GI	gastrointestinal
GLS	global longitudinal strain
GLDH	glutamate dehydrogenase
GM	Goeppert-Mayer (unit of two-photon absorption cross-section)
GNN	graph neural network
GPx4	glutathione peroxidase 4
GSH	glutathione
GSDMD	gasdermin D
HILIC-ICP-MS	hydrophilic interaction liquid chromatography–inductively coupled plasma mass spectrometry
HPLC-ICP-IDMS	high-performance liquid chromatography–inductively coupled plasma–isotope dilution mass spectrometry
HR	hazard ratio
hs-cTnI/T	high-sensitivity cardiac troponin I/T
IC50	half-maximal inhibitory concentration
ICP-MS	inductively coupled plasma mass spectrometry
IDMS	isotope dilution mass spectrometry
IMM	inner mitochondrial membrane
IND	Investigational New Drug (application)
KEAP1	Kelch-like ECH-associated protein 1
Ki	inhibitory constant
KIM-1	kidney injury molecule-1
LC3-II	microtubule-associated protein 1A/1B-light chain 3-II
LOQ	limit of quantification
LVEF	left ventricular ejection fraction
MCL-1	myeloid cell leukaemia sequence 1
MDR1/P-gp	multidrug resistance protein 1/P-glycoprotein
MIBI	methoxyisobutylisonitrile
3MLCT	triplet metal-to-ligand charge transfer
MLKL	mixed lineage kinase domain-like protein
MMR	mismatch repair
MOF	metal–organic framework
MOMP	mitochondrial outer membrane permeabilization
MPP	mitochondria-penetrating peptide
mRCC	metastatic renal cell carcinoma
MTD	maximum tolerated dose
MTMC	mitochondria-targeting metal complex
mtDNA	mitochondrial DNA
MTS	mitochondrial targeting sequence
MW	molecular weight
NADH	nicotinamide adenine dinucleotide (reduced form)
NADPH	nicotinamide adenine dinucleotide phosphate (reduced form)
NCS	nerve conduction studies
NER	nucleotide excision repair
NfL	neurofilament light chain
NGAL	neutrophil gelatinase-associated lipocalin
NHC	N-heterocyclic carbene
NHE	normal hydrogen electrode
NIR	near-infrared
NRF2	nuclear factor erythroid 2-related factor 2
NSCLC	non-small-cell lung cancer
NT-proBNP	N-terminal pro-B-type natriuretic peptide
OCR	oxygen consumption rate
OMM	outer mitochondrial membrane
OS	overall survival
OXPHOS	oxidative phosphorylation
PBMC	peripheral blood mononuclear cell
PDK	pyruvate dehydrogenase kinase
PDO	patient-derived organoid
PDT	photodynamic therapy
PDX	patient-derived xenograft
PET	positron emission tomography
PFS	progression-free survival
phen	1,10-phenanthroline
PINK1	PTEN-induced kinase 1
PK	pharmacokinetics
PK/PD	pharmacokinetic/pharmacodynamic
PLIM	phosphorescence lifetime imaging microscopy
PR	partial response
PRO	patient-reported outcome
PS-MOF	porphyrin-sensitised metal–organic framework
RES	reticuloendothelial system
RNA-seq	RNA sequencing
ROS	reactive oxygen species
RP2D	recommended Phase 2 dose
SD	stable disease
SEC-ICP-MS	size-exclusion chromatography–inductively coupled plasma mass spectrometry
SI	selectivity index
SPECT	single-photon emission computed tomography
SS-31	Szeto–Schiller peptide 31
TCA	tricarboxylic acid (cycle)
TDM	therapeutic drug monitoring
TMRM	tetramethylrhodamine methyl ester
TNBC	triple-negative breast cancer
TPP+	triphenylphosphonium cation
TrxR	thioredoxin reductase
TrxR1	thioredoxin reductase 1 (cytosolic)
TrxR2	thioredoxin reductase 2 (mitochondrial)
TTE	transthoracic echocardiography
UHPLC-ICP-MS	ultra-high-performance liquid chromatography–inductively coupled plasma mass spectrometry
UHPLC-SEC-ICP-MS	ultra-high-performance liquid chromatography–size-exclusion chromatography–inductively coupled plasma mass spectrometry
UCP	uncoupling protein
ULN	upper limit of normal
UPLC-MS/MS	ultra-performance liquid chromatography tandem mass spectrometry
XPC	xeroderma pigmentosum complementation group C
z-VAD-fmk	carbobenzoxy-valyl-alanyl-aspartyl-[O-methyl]-fluoromethylketone (pan-caspase inhibitor)
ΔΨm	mitochondrial membrane potential
ΔΨp	plasma membrane potential
ΦΔ	singlet oxygen quantum yield
